# Estimating food production in an urban landscape

**DOI:** 10.1038/s41598-020-62126-4

**Published:** 2020-03-20

**Authors:** Darren R. Grafius, Jill L. Edmondson, Briony A. Norton, Rachel Clark, Meghann Mears, Jonathan R. Leake, Ron Corstanje, Jim A. Harris, Philip H. Warren

**Affiliations:** 1 The University of Sheffield, Department of Animal and Plant Sciences, Sheffield, S10 2TN United Kingdom; 2 Cranfield University, School of Water, Energy and Environment, Cranfield, Bedfordshire MK43 0AL United Kingdom; 3 University of Derby, College of Life and Natural Sciences, Derby, DE22 1GB United Kingdom; 4 The University of Sheffield, Department of Landscape Architecture, Sheffield, S10 2TN United Kingdom

**Keywords:** Ecosystem services, Urban ecology, Sustainability

## Abstract

There is increasing interest in urban food production for reasons of food security, environmental sustainability, social and health benefits. In developed nations urban food growing is largely informal and localised, in gardens, allotments and public spaces, but we know little about the magnitude of this production. Here we couple own-grown crop yield data with garden and allotment areal surveys and urban fruit tree occurrence to provide one of the first estimates for current and potential food production in a UK urban setting. Current production is estimated to be sufficient to supply the urban population with fruit and vegetables for about 30 days per year, while the most optimistic model results suggest that existing land cultivated for food could supply over half of the annual demand. Our findings provide a baseline for current production whilst highlighting the potential for change under the scaling up of cultivation on existing land.

## Introduction

Food security and agricultural sustainability are global issues of rising concern^1^. This is driven by the challenge of feeding a growing population from a finite and diminishing global soil resource^2,3^, and by the spatial disconnection between agricultural production and the urban systems in which an ever increasing proportion of that population live^4,5^. We expect changes at relatively local scales to be important in meeting these challenges^6^. The potential of own-grown urban food production (i.e. recreational or non-commercial gardening) as a local solution to a global problem has attracted increasing interest in recent years^5,7–15^, driven by a multitude of ecological, sustainability, social, recreational, therapeutic, mental and physical health, and well-being benefits to local areas and residents^3,11,14,16–35^.

Whilst urban agriculture and own-growing have attracted considerable interest and advocacy, systematic evaluation of their proposed benefits remains limited^13,36–39^. Quantitative estimates of actual and potential urban food production are key to such evaluations, but are rare^34,40–42^. This stems from both the relatively recent interest in the subject and the specific challenges associated with obtaining such estimates. Nonetheless if the role of city-wide urban food production is to be assessed, and its relationships to urban form and to other ecosystem services are to be understood^35,43^, such data are crucial.

Estimating urban food production is challenging, as it is spread over many highly fragmented and usually small growing spaces, managed by many different users growing a wide variety of often finely intermingled crops. Further, much of the consumption is direct, not passing through any transaction which would generate systematic records^40^. For example, in the United Kingdom, urban food production predominantly occurs in either private residential gardens or communally administered allotment sites. Allotments are generally managed by local authorities or organisations, with plots of land assigned to individuals to use for fruit and vegetable cultivation. The potential of produce from urban greenspaces other than gardens and allotments (e.g. fruit from trees, fungi, berries etc.) is also relatively unexplored^42,44,45^. Quantifying and understanding the potential of different types of urban landscapes to produce their own food is essential to understanding, and planning, urban sustainability scenarios.

Here we provide the first comprehensive estimate of potential food production in a UK urban landscape from land currently used for some form of food production: allotment sites, private residential gardens and urban fruit trees. Our study system comprises three neighbouring large towns in the UK Midlands (Bedford, Luton and Milton Keynes) which together represent a range of typical urban forms in the UK. We estimate food production on allotments and residential gardens based on: GIS-derived data for the total area of allotments and gardens across the three towns, survey data for the proportional areas of allotments^46,47^ and private gardens^48^ that are cultivated for food growing, and measured yields for common own-grown crops in the UK^46^. Estimates of fruit tree production combine surveys of fruit tree occurrence across all land uses in the study towns with yields measured from allotment fruit trees (see Methods). Holding fruit tree production, crop yield density, and total growing space constant, we vary the proportional cultivation intensity within gardens and allotments in three scenarios to estimate food production for the study area: (1) ‘conservative current’ assumes proportional allotment cultivation at the mean observed in surveys (52%), and garden cultivation only that proportion specifically recorded as being used for food growing in garden surveys (2%); (2) ‘full current’ uses the same allotment proportion, but includes all areas from gardens which were cultivated for unspecified purposes (i.e. could be food production – 20%); and (3) ‘maximum potential’ uses the allotment proportion targeted for food production by local authorities (75%, slightly less than the maximum observed in allotment surveys (88%)), and the maximum observed proportion of garden used for food production (30%).

## Results

**Figure 1:**
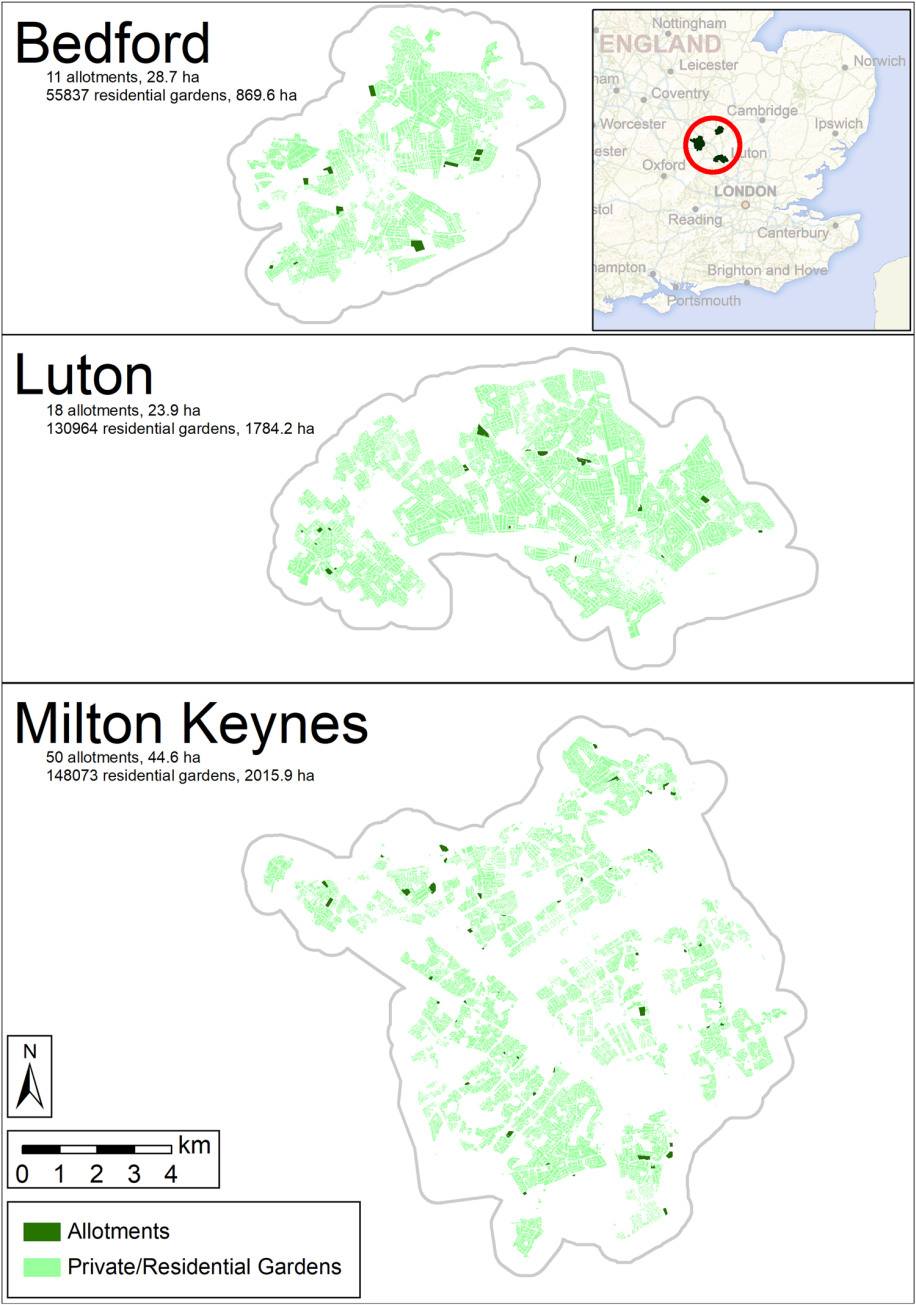
Study area with locations and distribution of allotment sites and private residential gardens in Bedford, Luton and Milton Keynes, UK. Overview map contains UK Ordnance Survey data © Crown copyright and database right (2019).

The study area contained 79 allotment sites (11 in Bedford, 18 in Luton and 50 in Milton Keynes), in total comprising an area of 97 ha. Private residential gardens constituted a total area of 4670 ha (870 in Bedford, 1784 in Luton and 2016 in Milton Keynes) (Fig. 1).

### Areas of allotments and gardens under cultivation

For allotments, the mean percentage of each plot under cultivation, derived from ground surveys in Leicester (see Methods), was 52%, and all such cultivation was modelled as food production. For residential gardens surveyed in Leicester, Cardiff and Oxford^48^, (see Methods for details), although most were cultivated, only 16.7% had areas recorded explicitly as food cultivation, and across all measured garden area the mean proportion confirmed used for food growing was less than 2%. However, for gardens where cultivation of any type occurred, proportional areas of potential production were much higher (Table 1).

**Table 1:** Summary data derived from garden surveys in Leicester, Cardiff and Oxford^48^ giving frequency and areas of garden either confirmed to be under cultivation for food production when surveyed, or recorded as having non-specific cultivated areas which could have been used for food production (these exclude cultivated areas confirmed to be non-food producing at the time of survey, e.g. flower beds).

	Number of gardens	Frequency (% of gardens)	Total surveyed area (m^2^)	Mean % area for all gardens	Mean % area for only gardens with either type of cultivation present	Mean, range & SD area (m^2^)
Confirmed food crop cultivation	26	16.7%	529.4	1.9%	10.1%	3.4 0–69 11.2
Non-specific cultivation	144	92.3%	5516.0	19.6%	21.9%	35.6 0–188 36.9
Total surveyed	156	—	28083.8	—	—	180.0 14–1084 140.5

### Estimated food production

**Figure 2:**
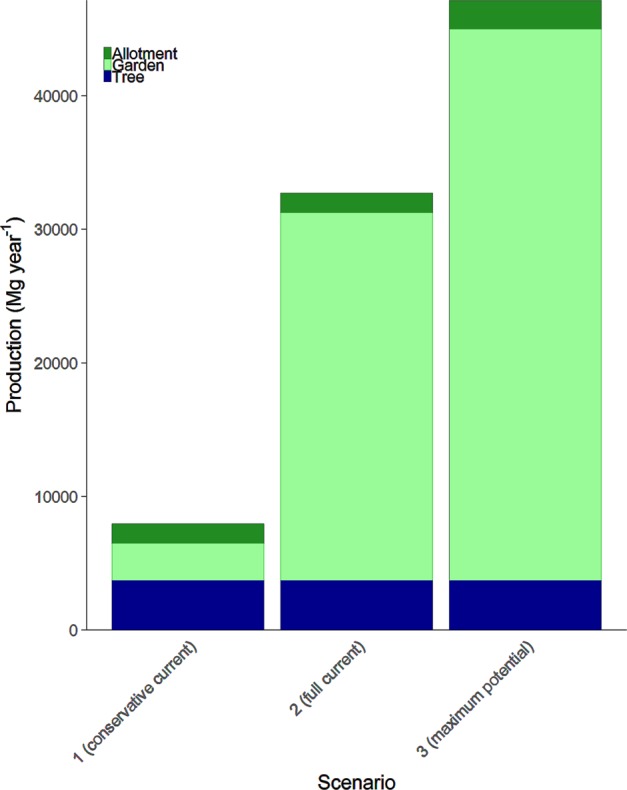
Potential allotment, residential garden and fruit tree food production (Mg year^−1^) in Milton Keynes, Bedford and Luton, UK, under current and potential scenarios.

The allotment and garden areas under confirmed or possible cultivation for food were combined with data for mean crop mixes and crop-specific yields per unit area (see Methods) to generate estimates of total production across the urban areas for each of the three different scenarios (Fig. 2). The conservative current scenario (1) estimates that the study area’s allotments and gardens are capable of producing 4240 Mg of food per year (25^th^ and 75^th^ percentiles: 3700, 4670); the full current scenario (2) estimate is 28,990 Mg year^−1^ (25^th^ and 75^th^ percentiles: 25,290, 31,930); and the maximum potential scenario (3) estimate is 43,400 Mg year^−1^ (25^th^ and 75^th^ percentiles: 37,860, 47,800).

Across various types of greenspace, approximately 1% of individual recorded trees were of four commonly eaten fruit-producing species: apple, plum, pear and damson. On this basis, estimated edible fruit production by urban trees totalled 3710 Mg year^−1^; slightly less than that for potential allotment and residential garden production under the conservative current scenario. Broken down by land-use, the majority of this production is in residential areas, followed by intensively used green spaces (e.g. public parks, cemeteries) (see Supplementary Table S2).

When combined with potential production from allotments and residential gardens, urban fruit trees considerably augment potential food provision in the study area (Table 2, Fig. 2). Urban fruit tree production is not changed between scenarios, but it is easy to see the potential for increased production with even a small proportional increase of fruiting species in the urban tree mix.

**Table 2:** Total potential urban food production (Mg year^−1^), production density and per capita production in Milton Keynes, Bedford and Luton UK, under current and potential scenarios.

Scenario	Mean Production (Mg year^−1^)	25^th^ percentile	75^th^ percentile	Mean Production Density (kg ha^−1^ year^−1^)	Mean Production per Capita (kg capita^−1^ year^−1^)
1	7950	6470	9330	435	13.4
2	32710	28070	36580	1787	55.0
3	47120	40640	52450	2575	79.2

### Implications for food supply and demand

Food production is one of the key ecosystem services upon which we depend and, although typically not given prominence in discussion of urban ecosystems, it is important to understand the potential it might have to contribute to human well-being in such environments. Despite this, estimates of the importance of urban food production are rare^40,42^. Here we use a variety of directly derived data for crop production, crop mixtures, cultivated areas, tree species and land-use to derive comprehensive estimates of current, and potential, fruit and vegetable production for three urban areas in the UK. Current production (scenario 1) is modest, though far from trivial, representing approximately 13.4 kilograms of edible produce per person in the urban population per year (Table 2). In context, the UK government recommends consumption of about 146 kg of fruit and vegetables per person per year (five 80 g portions per person per day^49^). Our estimated current production clearly represents only a small part of this (about 9%) or, put another way, the estimated production under scenario 1 would be sufficient to supply the population of our study area (594,899) for about 33 days at the recommended diet rate. Alternatively, this amount of production could meet the annual fruit and vegetable demand of approximately 54,500 people.

The above figures are based on an assumption of 100% edibility of harvested crops. Although true for many of the most widely-grown crops such as potatoes, some crops cannot have their total recorded harvest weight consumed. To provide a lower bound to our estimates, we consider a broad multi-crop mean edible fraction of 0.869^43^. This assumption lowers the estimate for scenario 1 to 11.6 kg per capita per year; enough to supply the study area population’s fruit and vegetable demand for circa 29 days, or 47,300 people for a year.

Although there are very few similar studies with which to make comparisons, our estimate here seems broadly consistent with such others as there are. CoDyre *et al*.^40^, studying domestic and community/allotment gardens, in Guelph, Canada, estimated that about 2% of the city’s requirements could be supplied at current cultivation and production rates, with an average garden producing enough to supply an adult with recommended amounts of fruit and vegetables for about 32 days. An average yield of 14.3 Mg ha^−1^ year^−1^ was found, compared to our observed figure of 29.5 Mg ha^−1^ year^−1^. Edmondson *et al*.^46^ estimated that current own-grown production in Leicester feeds approximately 2.6% of the city population’s fruit and vegetable recommendations. Other studies suggest similarly modest contributions to overall food requirements^12,21,50,51^, but comparisons are not straightforward as the methods, horticultural approaches considered, and types of food produced differ among these studies. This lack of data also complicates comparisons between potential urban and/or non-commercial gardening and rural commercial gardening, however some findings suggest comparable yield efficiencies across numerous crops^46^.

Only a few studies, including this one and those of CoDyre *et al*.^40^ and Edmondson *et al*.^46^, are based on assessing estimated production for sites currently used for food growing. Most other studies address the interesting, but slightly different, question of what potential food production might be if one or more particular types of urban land were given over to food growing, or how much land and under what types of cultivation (conventional, intensive, hydroponic, etc) would be required to feed a city’s population – and whether this land is available^12,21,42,50,51^. Despite the differences in approach, at the ‘lowest’ levels of land availability, proportional cultivation, and typical productivity investigated, the conclusion that cities in developed countries are far from being self-sufficient in food production is clear. Additionally, any increase in urban agriculture would likely have to consider numerous barriers, such as the changing of entrenched social habits. However, what also emerges from past research and our own, is that there is considerable potential to increase the proportion of food contributed by urban production if these challenges can be met.

Some of this ‘potential’ may, in practice, already be being realised. Our scenarios 1 and 2 both represent the current situation (with the assumption that the cities used to estimate domestic garden areas cultivated for food growing were applicable to our study area), but span a range of uncertainty in the input data, and on this basis alone found a fourfold difference in estimated production. The difference between scenarios 1 and 2 is whether only the proportion recorded as food crop land in residential gardens is considered (scenario 1) or whether all cultivated garden land that currently might be growing food (i.e. not explicitly recorded as hosting non-food crops) is considered (scenario 2). Reality is probably somewhere between these two, but the difference highlights both the need for better data and the potentially large effect of even relatively modest changes in decisions about what to grow, even without any change in the currently cultivated area. The change between scenario 1 and scenario 2 takes the estimate of the number of days’ supply to the area (see above) up from 33 to 137 days per year.

Scenario 3 represents a potential future state, scaling cultivation in domestic gardens to the highest observed proportion in the data, and allotments to a widely used local authority guideline as a representation of a possible maximum state, with a resulting estimate of 198 days of local fruit and vegetable supply per year. Each successive scenario suggests a considerable increase in potential food production, despite the fact that they are based only on existing allotments and gardens, and in the case of the latter only involve food growing on already cultivated areas (i.e. not conversion of lawn or other non-cultivation land-uses). Altogether, the scenarios highlight an extraordinary potential for urban food production and sustainability, albeit in contention with the considerable challenge of altering the behaviour of urban residents to change how they manage their private gardens, how they utilise allotments and how they forage from public trees.

The different scenarios used here do not involve any change in fruit production from trees; all scenarios use the estimated current production, as we had no equivalent, data-based, rationale for alternative scenarios. However, the inclusion of production estimates for existing urban fruit trees highlights their significant potential: under the conservative current scenario, trees account for nearly half of potential production. Of course, relatively little of this resource may currently be subject to exploitation^45,52,53^, so fruit trees may represent a significant but largely untapped resource. Of the total recorded tree species here, 20% produce readily edible parts, with many additional species producing useful products that require additional processing (e.g. grinding into flour, extracting sap for syrup, cooking into preserves). Given that between 0% and 5% of tree stems, depending on land-use, were of the edible species for which data were available (see Supplementary Table S2), there is considerable potential to increase fruit production by even relatively small alterations in the ratio of fruit to non-fruit species planted in urban greenspace. A doubling of the number of fruit trees in all recorded land-uses would increase fruit production from this source to 7430 Mg, taking the total production under scenario 3 to 50,830 Mg, and this would still represent only a modest proportion of total urban tree stems (8%). The potential for urban fruit production under more extensive schemes is illustrated by a US study of urban apple trees^42^ showing that under the most ambitious planting and yield scenario considered it would be possible to supply 108% of the city population’s daily minimum recommended serving of fruit. Additionally, fruit trees bring many of the additional benefits attributed to trees more generally which include a variety of community and ecosystem services beyond food production alone^44^.

## Discussion

We have estimated food production in three UK towns under three scenarios, drawing on field-derived production and distribution data. We focused here on the capacity to produce food on existing land already at least partly cultivated for this purpose, and incidental production from trees growing as part of the wider urban green infrastructure. What we do not consider is the scope for increasing the extent of urban food growing space, either by conversion of other types of greenspace to food producing areas or by food growing in non-green spaces, such as on, or in, buildings (e.g. rooftop farming) which may have greater importance in more densely developed urban landscapes. Some food production studies have focused on these spaces^12,21,50,51^, with varying conclusions about the potential supply of food (variously: vegetables, fruit, poultry, eggs and honey) which could be produced, ranging from figures around 1% or less, up to values of 50% or more, depending on a range of assumptions about land-uses converted, crops grown, and efficiency of production. We also do not address the ways in which developments in agricultural science and technology may improve yields and efficiency in the future, as these remain unknowns with no supporting data upon which to base assumptions. A key next step for assessing urban food production potential would be to bring together the type of ground-based investigation described here, with work on urban land use change to develop more detailed and fine-grained scenarios for assessing urban food production potential. In doing so, we believe the possibilities for increasing the sustainability and resilience of urban food production to be considerable.

As data availability develops it may become increasingly possible to conduct quantitative cost/benefit comparisons of productivity^54^ and risk between urban and rural food production. Case-by-case consideration may be required, as situations vary with respect to the risks that urban food production can place on the local environment through chemical use^55^ and in turn the risks that historic urban industrial pollutants may place on food safety^56^. In the UK, the recreational rather than subsistence-based nature of most urban gardening may reduce pressure to employ potentially harmful chemicals in the gardening process, although some pesticide and fertiliser use is not uncommon^57^. Contaminants remaining from historic industrial pollution are generally of greater concern and can pose risks that necessitate soil testing and possible remediation^58^, while other urban soil conditions have been found comparable to or even less degraded than in traditional agricultural sites^34,59^.

There are also insights to be gained from history and cultural diversity. In a study of one UK city (Leicester), Edmondson^46^ found that allotment provision is currently only 16% of its peak of provision in the 1950s due to post-war food shortages. A return to an equivalent level for our study system would increase our estimate of total fruit and vegetable production to 30,200 Mg under scenario 1, and 274,990 Mg under scenario 3. The major effect of growing space provision on city-wide production emphasises both the potential for enhancing food production through infrastructure change, and supports recent interest in the importance of incorporating food policy into urban planning, despite its historical neglect^60,61^ (see also Supplementary Discussion for cultural diversity in urban agriculture). The future is uncertain in terms of how growers and governments may react to changing levels of food security concern, but it is possible to imagine a resurgence of government initiatives in the style of World War 2’s ‘Dig for Victory’ to encourage and support own-growers in increasing their own food production. Were this to happen, quantitative estimates such as we provide here will be useful in better understanding the potential of urban food production.

## Methods

### Study area

The project study area was the combined urban area of three large UK towns (Bedford, Luton and Milton Keynes; total cover 193 km^2^; Fig. 1). Together the towns exhibit a broad range of urban forms and histories, capturing much of the diversity found across the UK’s urban landscapes.

Bedford (52° 8’ N, 0° 27’ W) developed in the Middle Ages as a market centre and exhibits the radial development pattern typical to many British towns. In 2011, its population was 106,940 across 36 km^2^, with a population density of 2,971 inhabitants km^−2^ ^62^.

Luton (Luton/Dunstable conurbation; 51° 52’ N, 0° 25’ W) developed heavily during the nineteenth century as an industrial centre. Its urban pattern contains large industrial zones and residential ‘terraced’ housing. The region had a 2011 population of 258,018 across 58 km^2^, with a population density of 4,448 inhabitants km^−2^ ^62^.

Milton Keynes (here including Newport Pagnell and Bletchley; 52° 0’ N, 0° 47’ W) is a planned ‘new town’, developed during the 1960s. The town is structured around a grid of major roads designed for ease of automotive travel, and is characterised by large areas of public green space, consisting of parks and green areas bordering foot and cycle paths^63^. Milton Keynes had a population of 229,941 in 2011, across 89 km^2^ with a population density of 2,584 inhabitants km^−2^ ^62^.

### Growing space locations

We focused our analysis on three types of greenspace used for food production: (1) allotment sites, (2) private gardens associated with residential dwellings (domestic gardens), and (3) public or privately owned areas other than (1) and (2) with significant tree cover (not pictured in Fig. 1 to avoid clutter, but widespread throughout the study area; see Supplementary Methods 1 and Fig. S2). Inclusion of the latter was to account for fruit producing trees distributed more widely in the urban environment.

Allotment sites within each town were identified from aerial photography^64^. Allotment locations were subsequently verified by internet search (e.g. local authority confirming the presence of an active allotment), and later by verification against the newly released UK Ordnance Survey Open Greenspace dataset (Jaccard’s coefficient for dataset agreement = 0.76). Private residential gardens were identified from Ordnance Survey MasterMap data as land parcels and clipped in GIS to the built-up extents of the three towns to limit the analysis to urban gardens. Non-allotment or garden land with potential for fruit tree occurrence was taken from a 0.5 m resolution land cover map described in Grafius *et al*.^65^ and then classified by land-use using a combination of data from OpenStreetMap®, OS AddressBase® Plus and OS MasterMap® (see Supplementary Methods 1 for details).

### Crop yield and crop mix data

UK own-grown crop yield data collected in 2012–2013 were available through a citizen science initiative^46^. Data on a total of 240 harvests were received from participants, and the mean and standard deviation of harvested yield of each fruit, including tree grown, or vegetable crop across all growers was expressed in kg m^−2^ (calculated from total weight of crop harvested and area used to grow the crop: see Supplementary Table S1). These data were used to parameterise yield calculations for individual crops.

Data on the areas under cultivation for different crop types in individual allotment plots were collected in Leicester (n = 62 plots from 14 different allotment sites)^46^ and Sheffield, UK during 2014 (n = 38 plots from 8 allotment sites; unpublished research)^47^. Data from Sheffield were collected following methodology outlined in Edmondson *et al*.^46^. Both datasets showed broadly similar crop mixes, and so were combined by mean value and used to parameterise the proportions of each crop used in the model for both allotments and residential garden production (see Supplementary Table S1).

### Cultivated area data

Data on the cultivated proportion of allotments were available from both the above Leicester (mean plot size = 264 m^2^, mean cultivated proportion = 52%, range = 15% − 87%, n = 62) and Sheffield datasets (mean plot size = 300 m^2^, mean cultivated proportion = 27%, range = 6% - 65%, n = 37 plots from 8 allotment sites). However, only proportions from the Leicester data were used in our analysis as the topography of Leicester more closely matched that of our study area, whereas the hilly nature of Sheffield facilitates unusual allotment layouts and low cultivated proportions.

Unlike allotments, domestic gardens are used for many purposes other than food growing. To estimate the proportions of ground area used for food crops we used data from the Biodiversity in Urban Gardens (BUGS 2) project^48^. We used data on areas used for food, non-food and unidentified plant cultivation from detailed maps produced in 156 gardens in the three cities closest to our study area: Cardiff (n = 53), Leicester (n = 52) and Oxford (n = 51). From this we calculated statistics relating to the proportion of residential garden area that could be confirmed to be currently growing food crops, and those containing cultivated spaces with unidentified plants (Table 1). Fruit trees in residential gardens were not considered at this stage to avoid double-counting (see below). No significant relationship was present between garden size and proportion or likelihood of fruit/vegetable cultivation, although the smallest recorded garden confirmed to be growing food crops was 75 m^2^. While these data relate to towns outside our study area, they provide robust, field-derived data which are comparable and applicable to our study area. Analyses of garden features in the BUGS project^48^ indicate a strong commonality of garden features and structure across different UK cities, giving us confidence in the transferability of these data.

Data for public fruit tree occurrence (upright only, excluding hedges and shrubs) were derived from two combined field surveys carried out across the study area. Survey locations (0.25 km^2^ grid squares) were selected using a stratified random approach and included a representative variety of land-use categories. Within each a greenspace fragment and a regular stopping points along a 1 km transect sample were taken from which all trees were recorded and identified, and their locations categorised by land-use (see Supplementary Methods 2). Here we focused on fruit tree species for which yield data were available: apple (*Malus pumila*), pear (*Pyrus communis*), damson (*Prunus domestica ssp. insititia*) and plum (*Prunus cerasifera* and *Prunus domestica*).

### Modelling

The model used in this analysis was created using the modelling software GoldSim (www.goldsim.com). Potential yield for each crop type was implemented as a normal distribution based on the observed mean and standard deviation, enabling calculation of an overall mean production value in tonnes per hectare per year (Mg ha^−1^ year^−1^), as well as 25^th^ and 75^th^ percentile values based these distributions, when all crops were combined. This calculated production potential had a mean of 29.5 Mg ha^−1^ year^−1^, a 25^th^ percentile of 25.7 Mg ha^−1^ year^−1^ and a 75^th^ percentile of 32.4 Mg ha^−1^ year^−1^.

Subsequent analysis was conducted in ArcGIS to calculate the total area of allotments and residential gardens across the entire study area, which were then adjusted by their respective cultivated proportions and multiplied by the above areal yield results to produce predictions of total crop production across the study area (see below). This enabled the calculation of total potential food production under current and possible scenarios^40^.

### Production scenarios

Three scenarios were used to explore the potential own-grown food production of the study area, and by extension its ability to meet the fruit and vegetable needs of its population (Table 2). Land availability, tree occurrence and crop yield efficiency were kept constant in all three scenarios, with only the cultivated proportion of currently available land adjusted. This was done in order to consider the potential impact of individual gardening habits and enthusiasm on city-wide food production, irrespective of broader changes in land-use or crop yield beyond the immediate control of individual gardeners. Calculations focus on total available land rather than a per-garden basis in order to maintain a broad view of city-wide production potential.

Scenario 1 represents a ‘conservative current’ scenario that bases potential food production on only the amount of current food crop cultivation that can be confirmed. The proportion of total residential garden area devoted to food production was limited to the area determined in the data to be growing food (land-use listed as vegetables or fruit; 2%). Allotment growing area was set to the mean observed value in mapping data from Leicester allotments (52%).

Scenario 2 represents a ‘full current’ scenario, which depicts the maximum potential food production under current cultivation levels. Here, the proportion of residential garden area under food cultivation was based on all areas confirmed to be growing food crops (as in Scenario 1), with the addition of spaces recorded as cultivated but with unidentified plants (cover recorded as ‘cultivated’ and land-use listed as unknown or ‘null’, 20%). This uncertainty meant these spaces were unable to be ruled out from producing food and were thus included as possible food production areas, representing a range of uncertainty in current production.

Scenario 3 represents a ‘maximum potential’ state under current land availability, in which all current residential gardens and allotments were assumed to be cultivated to a maximum feasible proportion of their land area. Here the productive proportion of total residential garden area is based on the maximum observed food-cultivated proportion in the data (30%). Allotment production was set to a common target value for cultivated area per plot given by many local authorities e.g.^66,67^ owning and managing allotments (75%), representing the maximum likely cultivated area beyond that set aside for access, storage, composting, and plot boundaries. Although this was believed to be unrealistically high in most cases, the maximum observed cultivated proportion of an allotment was higher at 88%^46^. This represented a unique situation of low crop diversity and intensive production unlikely to be replicated in most cases, so 75% was chosen as a target believed to be high but demonstrably attainable.

### Fruit tree production on other land

Potential food production from urban fruit trees was modelled independently of the above scenarios, as no data were available upon which to base different scenarios. Fruit tree production was instead calculated for each land-use category in which fruit trees were recorded (see above). This included commerce (e.g. office and retail), education, industrial, public services facilities, residential, transport facilities, extensively used green space (e.g. forest and grassland), intensively used green space (e.g. parks), other unclassified green space, and unclassified. Fruit tree yields were calculated in the same manner as other food crops; based on distributions of recorded yields, making it possible to calculate mean yield as well as 25^th^ and 75^th^ percentiles. Fruit tree production estimates were totalled and added to the total residential garden and allotment food production estimates in each scenario, in order to produce an overall urban food production estimate for each scenario.

## Supplementary information

Supplementary Information.

## Data Availability

Any data not included in the Supplementary Information is available upon request from the corresponding author.
