# Supplementary material for: Estimating food production in an urban landscape
Source: Sci Rep. 2020 Mar 20;10:5141. doi: 10.1038/s41598-020-62126-4 (PMC7083843; doi:10.1038/s41598-020-62126-4)
Supplement: Supplementary file 1 — Supplementary Information. [file 41598_2020_62126_MOESM1_ESM.pdf]

Estimating food production in an urban landscape - Supplementary materials

Darren R. Grafius, Jill Edmondson, Briony A. Norton, Rachel Clark, Meghann Mears, Jonathan Leake, Ron Corstanje, Jim A. Harris and Philip H. Warren

Supplementary Discussion: Additional background and literature review: own-growing in developing vs. developed countries

In developing countries, urban agriculture is often a necessity for subsistence, and evidence from data on land use and numbers of own-growers indicates it occurs at a significant scale[1–7]. In developed countries these secondary measures are less well documented, and in both contexts estimates of actual food production across entire urban landscapes are scant. Martellozo et al.[8] approach the problem at a very broad scale, using global estimates of urban areas and vegetable production to derive estimates of the proportion of current urban area necessary for urban agriculture to feed current urban populations. Aside from this, the few attempts to provide estimates of current or potential food production in urban areas follow a similar methodology: combining remote-sensed or mapped data on suitable land use, with estimates of per unit area production, under various types of cultivation[9–16]. Of these, two[10,13] assess sub-areas within a city while the others are at city scale; and all, apart from Hara et al.[10], are from the USA or Canada. The methods vary a great deal between studies, with each focusing on different mixes of land-use/cover types and suitability criteria. Crop mixes and yield estimates also vary, the latter often sourced from agricultural data. Despite these differences, there is some congruence in the broad conclusions that, while current levels of urban food production are modest, there is considerable potential to increase production using available (i.e. currently unbuilt) land. While these results are encouraging regarding the potential for urban food production, the differences in studied land types, high variability in yield estimates and data constraints underscore the need for expanding this evidence base. In this study, we provide, for the first time, a holistic estimate of urban food production for three urban areas in the UK, which incorporates various land types, with data on crop mixes, yields and cultivated areas derived from direct survey data.

Supplementary Methods 1: Detailed description of land-use mapping for public fruit tree classification

Public fruit tree occurrence was taken from a 0.5 m resolution land cover map described in Grafius et al.[17] (Figure S1). A hierarchical land-use map was then used to classify trees into their surrounding land-use categories. This map was generated for the study area combining three secondary data sources: OpenStreetMap® (downloaded March 2017; older versions not available; https://www.openstreetmap.org/copyright), OS AddressBase® Plus (October 2016 release) and OS MasterMap® (December 2013 release) data. OpenStreetMap land-use data were not spatially complete for the study area, but were used preferentially to classify areas. For areas of missing data, AddressBase® address classes (e.g. residential, commercial) were used to classify MasterMap topography area polygons with a centroid within 50 m of an address. Finally, MasterMap was used to assign a classification to remaining areas where possible. The resulting map consisted of 54% of

area classified as primarily built use (8 subclasses), 20% primarily green use (4 subclasses), 3% transport infrastructure, 2% water, 1% other and 20% not classifiable using secondary data sources (Figure S2).

Supplementary Methods 2: Detailed sampling methodology for public fruit trees

A 500 m mesh grid was placed across the entirety of each urban area, and each 500 × 500 m grid square (hereafter 'tile') was classified into one of 25 urban form classes according to (a) percentage building cover (five categories), and (b) percentage cover of vegetation over 0.5 m tall (five categories). Five representatives of each urban form class were randomly selected, where available, yielding a total of 112 survey tiles. Within 78 survey tiles, a greenspace 'fragment' covering all land uses, public and private, was selected using a stratified random approach based on urban form. Within each fragment, the species/size composition and abundance of all trees were estimated using a combination of transects and complete surveys across the whole site, or sub-areas selected using a stratified random approach, to represent the range of land uses present.

Separately, in all 112 tiles, a 1 km (not straight line) transect was walked along public rights of way, sampling as wide a variety of the land uses present as possible (given access constraints). Due to these constraints, 9 transects were between 200-947 m in length and 9 were between 1052-1218 m in length. All other transects were 1km in length, +/- 50 m. As in the fragment sites, the composition and abundance of upright tree species (excluding hedges and shrubs) were recorded in a 10 m radius around each of eight equally spaced stopping points on the transect. The centre of the survey area was always on public routes, but the surveys extended into both public and private adjacent land.

Both sets of samples (fragments and transect stopping points) were classified according to the land-use in which they occurred, and used to separately estimate the proportional composition of studied fruit tree species for areas of tree cover within each land-use type across the study area. The two data sets yielded broadly similar estimates of fruit tree species proportional abundance, although the transect samples had slightly lower estimates of overall fruit tree abundance (0.67% of all recorded transect trees were studied fruit tree species, compared to 0.72% for fragment trees). We thus combined the proportional abundance of study species for both datasets by mean value into a single dataset of proportional tree occurrence by land-use type.

Study species accounted for 10% of total surveyed species (by number of species) producing parts that are edible with minimal preparation, but are believed to be the most well-known, recognisable and frequently managed for food crops. They are therefore expected to have a disproportionately high importance and recognisability in foraging circumstances, so production estimates for non-garden fruit trees were calculated for these species. Some other species producing edible components had high occurrence but were excluded from analysis on the basis of being deemed less relevant to potential foraging as a direct food source (e.g. sloe *Prunus spinose*; wild cherry *Prunus avium*; and hazel *Corylus avellana*).

Abstract for Clark, Rachel (2014), "Investigating land use on Sheffield's allotments: Potential for food production and provision other services", Research Thesis, University of Sheffield, Department of Animal and Plant Sciences[18]

This study uses a multidisciplinary approach to explore the land use on 38 allotment plots in Sheffield, involving mapping plots to quantify the proportion cultivated and questionnaires to obtain details of management practices and motivations of plot holders. Cultivated land occupied on average 27% of the total plot area, ranging widely from 6% to 67%, driven predominantly by the tenancy length under the current plot holder, and decreasing with more people sharing the plot. Produce grown within plots was diverse, the most prevalent being crops such as potatoes, onions, leeks and green beans which occupied a relatively large proportion of the growing area. Evidence for substantial meal provision emphasises the need for more accurate quantification of the extent of food production on allotments. Plot holders reported the most important aspects of allotment management to be spending time outdoors and associated wellbeing benefits, and along with evidence for pro-environmental management practices this highlights the potential for involvement of allotment gardeners in bottom-up conservation schemes. This study highlights avenues for further investigation into allotments for food production and conservation of urban biodiversity, and emphasises the need for increased support from local authorities to fully utilise allotments as multi-functional green infrastructure.

Table S1: Mean and standard deviation of crop yields used in analysis[19], and mean relative proportions of cultivated crops[18] (mean of that used in Clark[18] and Edmondson et al.[19]).

Crop Type	Mean Yield (kg/m^2)	Yield SD (kg/m^2)	N	Mean Proportion of cultivation (%)
Asparagus	0.2	-	1	0.43%
Beetroot	1.1	0.5	5	2.12%
Blackberries	1.7	0.2	3	1.09%
Blueberries	1.3	-	1	0.50%
Borlotti beans	1.3	-	1	0.14%
Broad beans	2.8	2.4	6	2.93%
Brown onions	2.3	2.0	13	7.74%
Brussels sprouts	1.5	0.8	4	1.60%
Cabbage	1.8	1.4	8	3.12%
Carrots	1.1	1.3	4	1.60%
Cauliflower	0.8	0.6	3	0.53%
Celeriac	1.0	-	1	0.24%
Celery	0.5	-	1	0.11%
Chilli	2.0	-	1	0.18%
Climbing French beans	6.5	10.2	6	3.09%
Courgettes	6.0	6.6	8	4.96%
Cucumbers	7.1	7.8	4	0.49%
Currants	1.3	1.0	6	3.39%
Dwarf beans	0.5	-	1	1.54%
Fennel	0.3	-	1	0.17%
Garlic	0.5	0.4	4	2.88%
Globe artichoke	2.1	0.1	2	0.54%
Gooseberries	1.9	1.9	4	2.28%
Jerusalem artichoke	5.8	5.0	3	0.15%
Kale	2.1	2.0	2	1.26%
Leeks	2.3	1.4	8	4.80%
Lettuce	1.0	0.6	3	1.26%
Mangetout peas	1.0	0.6	4	0.08%
Pak choi	5.3	4.5	2	0.03%
Parsnip	2.4	1.3	6	1.46%
Potatoes	2.6	1.4	8	14.38%
Pumpkin	2.9	2.6	5	1.62%
Purple sprouting broccoli	0.2	0.3	2	2.57%
Raspberries	1.1	1.3	6	6.03%
Red cabbage	2.4	0.1	2	0.10%
Red onions	1.0	0.3	3	0.25%
Rhubarb	1.0	1.1	3	2.23%
Rocket	3.0	-	1	1.29%
Runner beans	3.3	2.2	5	4.43%

Scorzonera	3.7	4.4	2	0.05%
Shallots	3.2	2.7	4	0.42%
Spinach chard	14.3	-	1	1.26%
Spring onions	0.5	0.0	2	0.56%
Strawberries	1.0	1.2	3	4.95%
Sugar snap peas	0.8	0.8	3	3.28%
Swede turnips	2.2	2.5	4	0.91%
Sweet peppers	2.6	-	1	0.06%
Sweetcorn	0.7	0.5	5	1.89%
Tomatoes	5.1	4.2	6	3.01%
**Fruit trees**				
Apple	1.7	1.6	46	-
Damson	0.4	0.1	2	-
Pear	1.2	1.7	3	-
Plum	0.9	0.9	11	-

Table S2: Observed mean proportions of fruit-bearing tree species to all tree species (combined datasets) and potential urban non-allotment fruit tree food production (Mg year^{-1}) in Milton Keynes, Bedford and Luton, UK, by land-use type.

Land-use	Apple Proportion	Pear Proportion	Plum Proportion	Damson Proportion	Mean Production (Mg year^{-1})	25th percentile	75th percentile
**Agriculture**	0.0570	0.0133	0.0049	0	94.2	53.6	126.8
**Commerce**	0.0125	0	0	0	44.7	22.4	62.4
**Education**	0.0017	0	0.0017	0	8.7	6.1	11.3
**Industrial**	0.0103	0.0007	0.0301	0.0088	163.0	121.3	204.9
**Public services facilities**	0	0	0	0.0357	1.4	1.2	1.7
**Residential**	0.0113	0.0041	0.0448	0.0130	2,832.1	2,112.6	3,558.4
**Transport facilities**	0	0	0	0.0208	2.4	1.9	2.8
**Extensively used green space**	0.0007	0.0001	0.0038	0.0076	111.3	87.9	136.9
**Intensively used green space**	0.0074	0.0031	0.0170	0.0578	395.8	326.1	478.0
**Other unclassified green space**	0	0	0.0270	0	18.6	10.8	26.6
**Unclassified**	0	0	0	0.0182	41.2	33.1	48.0

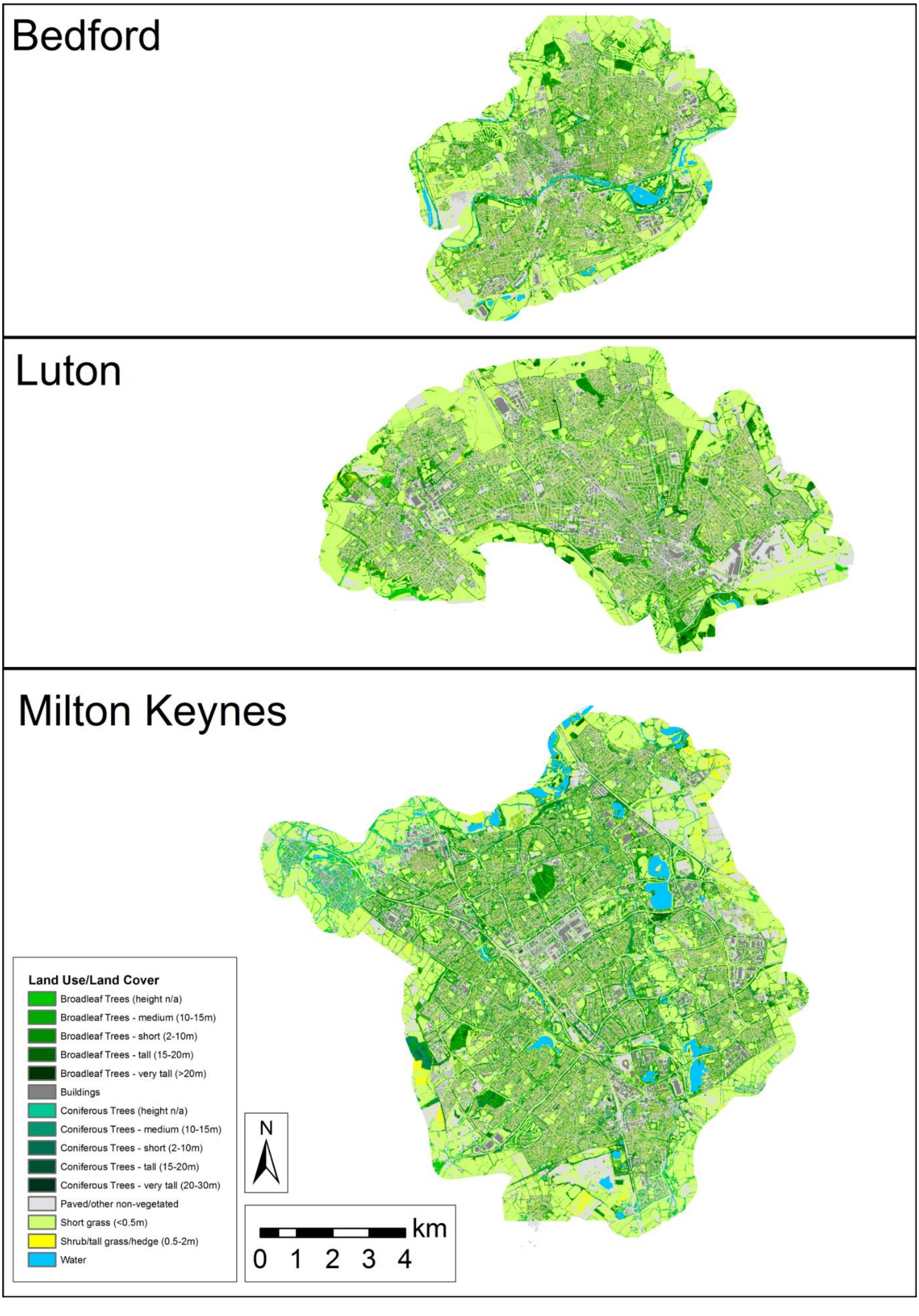

Figure S1: Land cover for Bedford, Luton and Milton Keynes, UK. Map created using ArcMap 10 and data from UK Ordnance Survey MasterMap and colour infrared aerial photography (described in detail in Grafius et al.[17]).

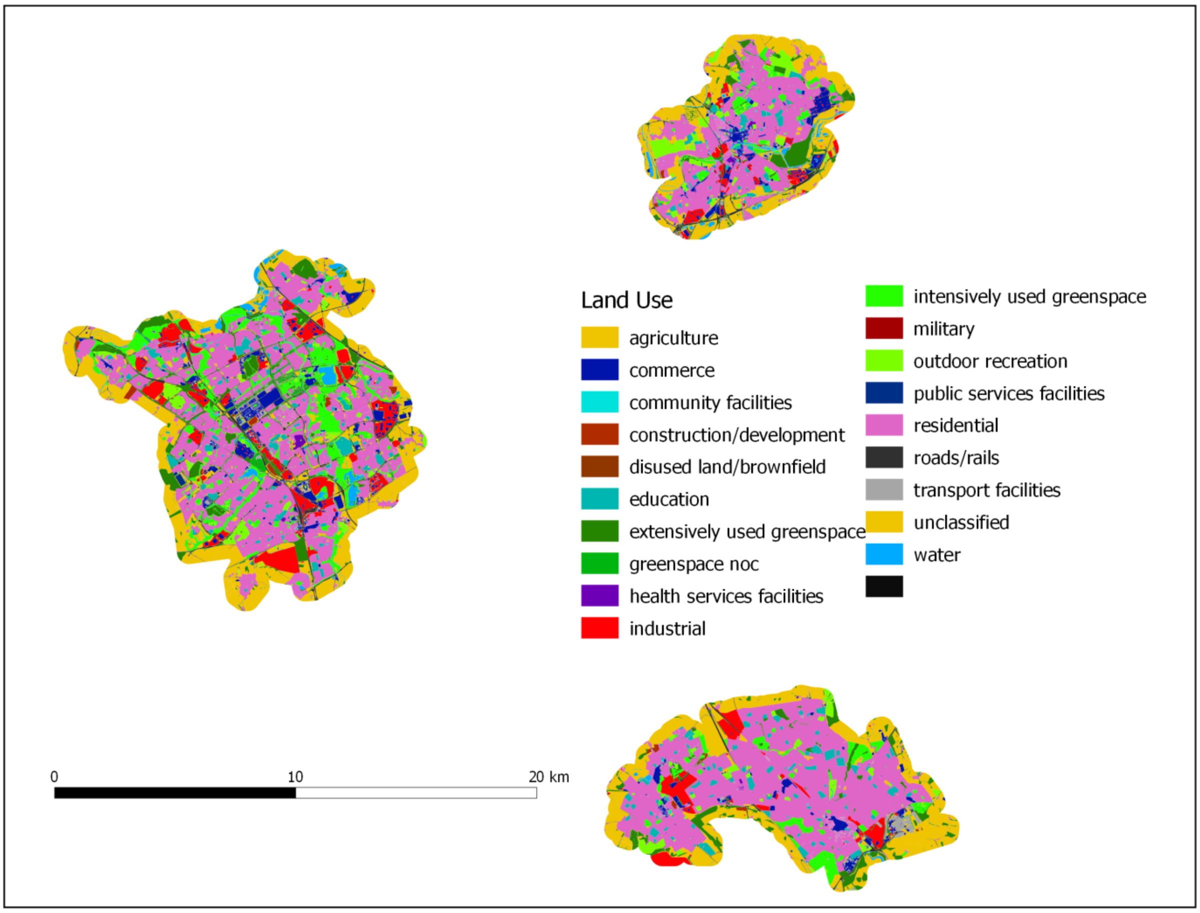

Figure S2: Land-use for Bedford, Luton and Milton Keynes, UK.

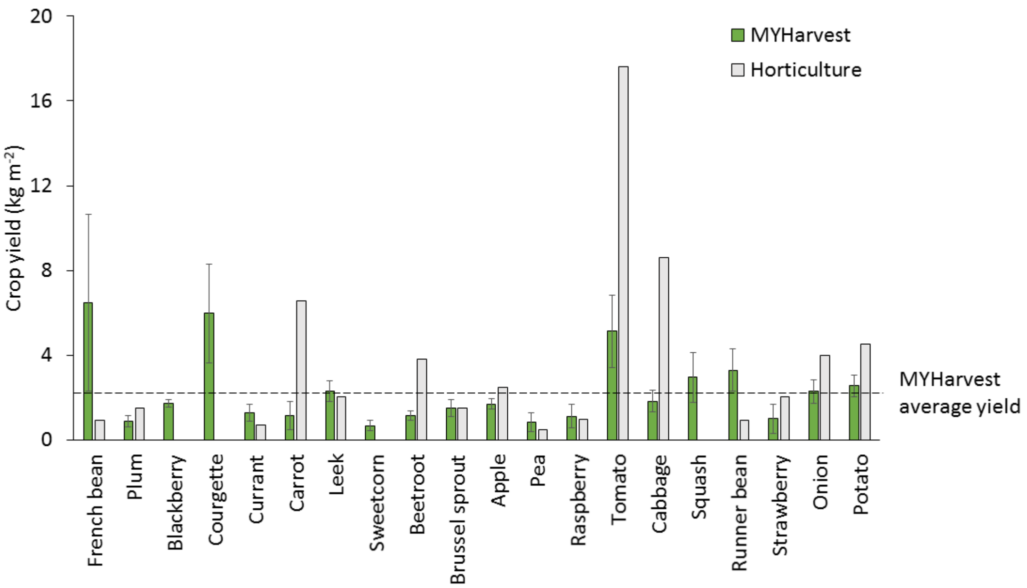

Figure S3: Comparison of own-grown crop yields measured by Edmondson et al.[19] against commercial yields for equivalent crops[20].

References

1. Madaleno, I. Urban agriculture in Belém, Brazil. *Cities* **17**, 73–77 (2000).

2. Lee-Smith, D. Cities feeding people: An update on urban agriculture in equatorial Africa. *Environ. Urban.* **22**, 483–499 (2010).

3. Eriksen-Hamel, N. & Danso, G. Agronomic considerations for urban agriculture in southern cities. *Int. J. Agric. Sustain.* **8**, 86–93 (2010).

4. De Zeeuw, H., Van Veenhuizen, R. & Dubbeling, M. The role of urban agriculture in building resilient cities in developing countries. *J. Agric. Sci.* **149**, 153–163 (2011).

5. Orsini, F., Kahane, R., Nono-Womdim, R. & Gianquinto, G. Urban agriculture in the developing world: a review. *Agron. Sustain. Dev.* **33**, 695–720 (2013).

6. Hamilton, A. J. *et al.* Give peas a chance? Urban agriculture in developing countries. A review. *Agron. Sustain. Dev.* **34**, 45–73 (2014).

7. Bellwood-Howard, I., Shakya, M., Korbeogo, G. & Schlesinger, J. The role of backyard farms in two West African urban landscapes. *Landsc. Urban Plan.* **170**, 34–47 (2017).

8. Martellozzo, F. *et al.* Urban agriculture: a global analysis of the space constraint to meet urban vegetable demand. *Environ. Res. Lett.* **9**, 064025 (2014).

9. Grewal, S. S. & Grewal, P. S. Can cities become self-reliant in food? *Cities* **29**, 1–11 (2012).

10. Hara, Y., Murakami, A., Tsuchiya, K., Palijon, A. M. & Yokohari, M. A quantitative assessment of vegetable farming on vacant lots in an urban fringe area in Metro Manila: Can it sustain long-term local vegetable demand? *Appl. Geogr.* **41**, 195–206 (2013).

11. McClintock, N., Cooper, J. & Khandeshi, S. Assessing the potential contribution of vacant land to urban vegetable production and consumption in Oakland, California. *Landsc. Urban Plan.* **111**, 46–58 (2013).

12. Ackerman, K. *et al.* Sustainable food systems for future cities: The potential of urban agriculture. *Econ. Soc. Rev. (Irel).* **45**, 189–206 (2014).

13. Ghosh, S. Measuring sustainability performance of local food production in home gardens. *Local Environ.* **19**, 33–55 (2014).

14. CoDyre, M., Fraser, E. D. G. G. & Landman, K. How does your garden grow? An empirical evaluation of the costs and potential of urban gardening. *Urban For. Urban Green.* **14**, 72–79 (2015).

15. Richardson, J. J. & Moskal, L. M. Urban food crop production capacity and competition with the urban forest. *Urban For. Urban Green.* **15**, 58–64 (2016).

16. Clark, K. H. & Nicholas, K. A. Introducing urban food forestry: A multifunctional approach to increase food security and provide ecosystem services. *Landsc. Ecol.* **28**, 1649–1669 (2013).

17. Grafius, D. R. *et al.* The impact of land use/land cover scale on modelling urban ecosystem

services. *Landsc. Ecol.* **31**, 1509–1522 (2016).

18. Clark, R. Investigating Land Use on Sheffield's Allotments: Potential for Food Production and Provision of Other Services (thesis). (University of Sheffield, 2014).

19. Edmondson, J. L. *et al.* Feeding a city – Leicester as a case study of the importance of allotments for horticultural production in the UK. *Sci. Total Environ.* **705**, 135930 (2020).

20. DEFRA. *Horticulture Statistics 2015*. https://www.gov.uk/government/statistics/horticulture-statistics-2015 (2015).
